# Clinical predictors of kynurenine pathway aberrations in schizophrenia and bipolar disorder

**DOI:** 10.1192/j.eurpsy.2022.927

**Published:** 2022-09-01

**Authors:** K. Skorobogatov, M. Leboyer, M. Foiselle, M. Morrens, L. De Picker

**Affiliations:** 1 Faculty of Medicine and Health Sciences, Collaborative Antwerp Psychiatric Research Institute (CAPRI), University Of Antwerp, Wilrijk, Belgium; 2 University Psychiatric Centre Duffel, Sinaps, Duffel, Belgium; 3 APHP CHU Mondor, Psychiatrie, Créteil, France

**Keywords:** kynurenine, schizophrénia, BIPOLAR, inflammation

## Abstract

**Introduction:**

Schizophrenia and bipolar disorder are severe mental illnesses that are known to have a considerable overlap in underlying pathophysiological mechanisms. More specifically, disturbances in the kynurenine pathway have been hypothesized as processes bridging altered immune responses and clinical manifestations of these illnesses.

**Objectives:**

The aim of this study was to investigate the abnormalities in serum kynurenine metabolites in schizophrenic and bipolar patients and the impact of clinical factors.

**Methods:**

Four patient groups were included in the current study: 1) Acute bipolar inpatients (n=205); 2) stable bipolar outpatients (n=116); 3) acute schizophrenia inpatients (n=111) and 4) stable schizophrenia outpatients (n=75); and one healthy control group (n=185). Clinical symptoms were established using symptom severity scales. The quantitative determination of serum kynurenine metabolites was performed using LC-MS/MS. General linear model and multivariate linear regression analyses were used to perform the statistical analysis with JMP Pro 15.

**Results:**

In line with previous research, the results indicate that serum kynurenine metabolites are disturbed in schizophrenic and bipolar patients compared to healthy controls. Whereas no differences were observed between schizophrenia and bipolar disorder, illness state and duration of illness clearly impacted kynurenine metabolite levels. Acutely ill patients had significantly lower levels compared to stable patients, which seemed to be driven by psychotic symptoms.

**Conclusions:**

To conclude, the results confirm the involvement of the kynurenine pathway in the pathophysiology of schizophrenia and bipolar disorder by lowered peripheral kynurenine metabolite level. In addition, an important role of acute psychotic symptoms and longer illness duration on these metabolite aberrances is demonstrated.

**Disclosure:**

No significant relationships.

